# DNA Barcoding Reveals Cryptic Diversity in *Lumbricus terrestris* L., 1758 (Clitellata): Resurrection of *L. herculeus* (Savigny, 1826)

**DOI:** 10.1371/journal.pone.0015629

**Published:** 2010-12-29

**Authors:** Samuel W. James, David Porco, Thibaud Decaëns, Benoit Richard, Rodolphe Rougerie, Christer Erséus

**Affiliations:** 1 Biodiversity Institute, Kansas University, Lawrence, Kansas, United States of America; 2 Canadian Centre for DNA Barcoding, Biodiversity Institute of Ontario, Guelph, Canada; 3 Laboratoire d'Ecologie, UPRES-EA 1293 ECODIV, FED SCALE, Bâtiment IRESE A, UFR Sciences et Techniques, Université de Rouen, Mont Saint Aignan, France; 4 Department of Zoology, University of Gothenburg, Göteborg, Sweden; Biodiversity Institute of Ontario, University of Guelph, Canada

## Abstract

The widely studied and invasive earthworm, *Lumbricus terrestris* L., 1758 has been the subject of nomenclatural debate for many years. However these disputes were not based on suspicions of heterogeneity, but rather on the descriptions and nomenclatural acts associated with the species name. Large numbers of DNA barcode sequences of the cytochrome oxidase I obtained for nominal *L. terrestris* and six congeneric species reveal that there are two distinct lineages within nominal *L. terrestris*. One of those lineages contains the Swedish population from which the name-bearing specimen of *L. terrestris* was obtained. The other contains the population from which the syntype series of *Enterion herculeum* Savigny, 1826 was collected. In both cases modern and old representatives yielded barcode sequences allowing us to clearly establish that these are two distinct species, as different from one another as any other pair of congeners in our data set. The two are morphologically indistinguishable, except by overlapping size-related characters. We have designated a new neotype for *L. terrestris*. The newly designated neotype and a syntype of *L. herculeus* yielded DNA adequate for sequencing part of the cytochrome oxidase I gene (COI). The sequence data make possible the objective determination of the identities of earthworms morphologically identical to *L. terrestris* and *L. herculeus*, regardless of body size and segment number. Past work on nominal *L. terrestris* could have been on either or both species, although *L. herculeus* has yet to be found outside of Europe.

## Introduction

*Lumbricus terrestris* L., 1758 occupies an important place in the nomenclature of earthworms, having been the first earthworm named [1], and has an important place in biological science and science education. It has been used in many studies of earthworm anatomy, behaviour, physiology and ecology, achieving the status of model organism long before this term came into common use in the last few decades. Virtually every student of biology in secondary or higher education systems of the Western world, as well as many places influenced by textbooks produced therein, has been presented the example of “*Lumbricus terrestris*” as an object of study. In some cases it may be doubted that *L. terrestris* was in fact on the dissection tray, but no matter; the name was used and biologists everywhere recall this as “the earthworm.” Darwin [2] referred loosely to earthworms in this way, though it is likely that one of the species whose activities he observed was *L. terrestris*. The species came into further prominence as an economic resource through the fish bait trade, notably in North America, where astonishing numbers are gathered from Canadian golf courses for domestic bait use and export to the United States of America [3]. Finally, *L. terrestris* is now considered an invasive species and has a prominent role in transforming soils and organic matter accumulations where it has invaded ecosystems previously devoid of earthworms, or replaced species with a comparable ecology [4], [5]. It routinely reaches population densities capable of consuming the entire annual leaf fall of north temperate deciduous forests, which is far more than above-ground herbivores normally do except in massive pest outbreaks [4], [6], [7].

With all the scientific, educational and popular attention devoted to *L. terrestris*, it is rather surprising to find that we do not really know what it is. Some of the problems stemmed from the brief original description and lack of a type specimen, but this was rectified in a detailed consideration of the nomenclatural history and identities of various earthworms cited as *L. terrestris*, with the designation of a neotype from the probable type locality in Uppsala, Sweden [8]. That neotype is now missing (The Natural History Museum, in litt.). Savigny [9], in describing *Enterion herculeum* Savigny, 1826 deposited a series of specimens which exists to this day. However, *E. herculeum* was later placed in the synonymy of *L. terrestris* [8], [10]. Richard et al. [11] detected two genetic clusters within nominal *L. terrestris*, reopening the debate. In this paper we revisit the question of the identities of the earthworms known as *L. terrestris* and *L. herculeus*, with the application of molecular and morphological data. We designate a new neotype for *L. terrestris* and provide a DNA barcode record for a syntype of *L. herculeus*. The latter is from Savigny's specimens, which were automatically syntypes because no holotype was fixed by the author. This barcoded specimen is here designated as the lectotype of *L. herculeus*.

## Materials and Methods

We examined 200 specimens of “*L. terrestris*” recently collected in Europe and North America, four specimens topotypic to the former neotype [8] of *L. terrestris* collected in Uppsala, Sweden in 1972; a syntype of *L. herculeus* and several specimens of five congeners: *L. castaneus* (Savigny, 1826); *L. centralis* Bouché, 1972; *L. festivus* Savigny, 1826; *L. friendi* Cognetti, 1904; and *L. rubellus* Hoffmeister, 1843 (Table 1). Morphological examinations were confined to nominal *L. terrestris* (including *L. herculeus*), including four specimens topotypic to the 1973 neotype of *L. terrestris*, six specimens from Parc du Chateau Brunoy, and five specimens collected in 2008 from Parc du Gally on the grounds of the Versailles Palace, the location of Savigny's material from the environs of Paris (M.B. Bouché, pers. comm., based on notes of Savigny). Tissue samples were obtained from three of the topotypic Uppsala *L. terrestris* collected in 1972, a syntype of *L. herculeus*, the five congeners, and the 198 recent specimens of “*L. terrestris*” one of which is the new neotype (GenBank HM388349; BOLD EW-ECO-0533) and topotypic to the former neotype of *L. terrestris*.

**Table 1:** Specimens included in the study.

Species	Country	Region	N
*Lumbricus castaneus*	France	Seine Maritime	9
	Andorra	Santa Julia	1
			10
*Lumbricus centralis*	France	Provence-Alpes-Cote d'Azur	1
*Lumbricus festivus*	France	Seine Maritime	8
	France	Ile de France	1
			9
*Lumbricus friendi*	France	Midi-Pyrenees	1
*Lumbricus rubellus*	France	Haute Normandie	9
*Lumbricus terrestris*	Canada	Ontario	63
	Denmark	Jutland, Arhus	1
	France	Languedoc-Roussillon	1
	France	Ile de France	7
	France	Haute Normandie	41
	France	Bretagne	2
	Norway	Nordland	1
	Norway	Hordaland	1
	Sweden	Jämtland	1
	Sweden	Scania	2
	Sweden	Småland	2
	Sweden	Uppland	2
	Sweden	Värmland	1
	Sweden	Västerbotten	1
	Sweden	Västergötland	2
	United States	Ohio	7
	United States	Iowa	8
			144
*Lumbricus herculeus*	Denmark	Jutland, Arhus	1
	France	Seine Maritime	24
	France	Ile de France	22
	Sweden	Scania	9
			56

The text references to lineages L1 and L2 correspond to *L. terrestris* and *L. herculeus*, respectively.

In total, 230 specimens from 6 species of *Lumbricus* were used for genetic examination of the divergence within the genus *Lumbricus* (Table 1). All these worms were processed for the campaign ‘Barcoding Earthworms’ (BCEW) at two different laboratories.

### Samples processed at Canadian Centre for DNA Barcoding

Lysis of the tissues was carried out in 50µl volume of lysis buffer and proteinase K incubated at 56°C overnight. DNA extraction followed a standard automated protocol on 96-well glass fiber plates (Ivanova et al. 2006). The 5′ region of COI used as a standard DNA barcode was amplified using M13 tailed primers LCO1490 and HCO2198 [12]. Failed samples from this first pass were amplified with a pair of internal primers combined with full length ones LepF1-MLepR1 and MLepF1-LepR1 [13]. A standard PCR reaction protocol was used for PCR amplifications and products were checked on a 2% E-gel 96Agarose (Invitrogen). Unpurified PCR amplicons were sequenced in both directions using M13 tails as primers. The sequencing reactions followed standard protocols of the Canadian Centre for DNA Barcoding (CCDB) [14], with products subsequently purified using Agencourt CleanSEQ protocol (Agencourt) and processed using BigDye version 3.1 on an ABI 3730 DNA Analyzer (Applied Biosystems).

The specimen from the type series of *Lumbricus herculeus* from the Savigny 1821 collection and the 1972 topotypic *L. terrestris* specimens were sampled for DNA (hereafter referred to as the museum specimens). The age and preservation of the specimens from which these tissues were sampled, however, demanded a different approach for extraction and amplification. Extraction was done manually with the Nucleospin tissue extraction Kits and PCR amplification was done with 6 pairs of primers in order to amplify overlapping fragments of about 160bp (Table 2). The same primers and the standard protocol of the CCDB [14] were used for the sequencing of those fragments.

**Table 2:** Primers used to obtain short overlapping barcode sequence fragments.

5′ - 3′	
**1st pair**	
LCO1490_t1	TGTAAAACGACGGCCAGTGGTCAACAAATCATAAAGATATTGG
EWLt-1R	CGCCAATRAAGACTGGTATYAC
**2nd pair**	
EWLt-2F	TTATACAATACAATCGTTACTGC
EWLt-2R	GAAACTARGAGAATAAGRGAGGG
**3rd pair**	
EWLt-3F	CATAAGATTTTGACTTCTRCC
EWLt-3R	AGRATAGAGGAYGCACCTGC
**4th pair**	
EWLt-4F	CTTGCCAGRAATCTCGCCCA
EWLt-4R	ACAAAYAGAGGGATTCGYTCTAG
**5th pair**	
EWLt-5F	TCCCTCCATTTRGCAGGKGC
EWLt-5R	GTTARGAGTATTGTGATTGCYCCK
**6th pair**	
EWLt-6F	AATYACAGTAGTYCTCCTYCTCCT
HCO2198_t1	CAGGAAACAGCTATGACTAAACTTCAGGGTGACCAAAAAATCA

### Specimens processed at University of Gothenburg

Twenty-four specimens morphologically identified as *L. terrestris* were collected in Scandinavia (21 in Sweden, two in Denmark, and two in Norway), in 2008–2009. DNA was extracted from a tissue sample of each worm with the QIAGEN DNeasy® Blood & Tissue Kit, after which PCR reactions were performed using the COI primers LCO1490 (forward) and HCO2198 (reverse) [12], the reverse primer sometimes replaced by COI?E [15]; all following standard protocols. PCR products were purified using an Omega E.Z.N.A. cycle-pure kit, and sent to Macrogen, South Korea, for sequencing.

### Sequence analysis

Sequences were assembled with Sequencer 4.5 (GeneCode Corporation, Ann Arbor, MI, USA) and aligned by eye using BIOEDIT version 7.0.5.3 [16]; we observed no indels in this coding region of the mitochondrial genome and therefore all base positions were aligned with confidence in positional homology. Distance analyses were conducted with MEGA4 [17] using a Neighbor-Joining [18] algorithm and distances corrected with the Kimura-2 parameter model [19]. The robustness of nodes was evaluated through bootstrap re-analysis of 1000 pseudoreplicates.

## Results

### Barcode data

230 full length barcodes were obtained ranging between 508 and 658 bp. From the syntype of *L. herculeus* we obtained 5 of the 6 fragments with 4 consecutive overlapping ones producing a continuous sequence of 480 bp. Only one of the 1972 Uppsala museum specimens of *L. terrestris* yielded a sequence, a 144 bp fragment. A complete COI barcode was obtained from the specimen collected in Uppsala in 2009 (the replacement neotype, GenBank HM388349; BOLD EW-ECO-0533). Sequences are publicly available on BOLD [20]; http://www.barcodinglife.org) within the project LTERH and in GenBank (Table S1).

**Figure 1:**
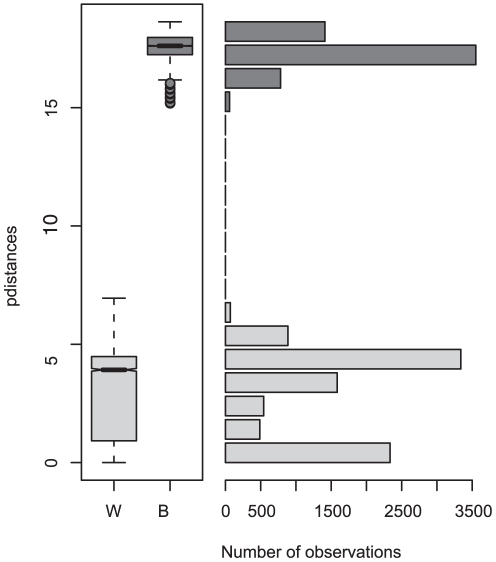
Boxplots of intraspecific (W; gray bars) and interspecific (B; black bars) genetic distances (K2P) for *Lumbricus* species with corrected taxonomy separating *L. terrestris* from *L. herculeus*. Each boxplot represents: the discarded outliers (external dots), the smallest and largest observations (external bars), the lower and upper quartiles (limits of the box) and the median (within-box black line). The boxplots are notched and indicate that medians differ if the notches do not overlap.

The mean intraspecific and interspecific variations for COI in the genus *Lumbricus* are 1.24% and 19.81%, respectively, except for nominal *L. terrestris* which exhibits the highest intraspecific value in the dataset at 8.93% and a range of 0% to 19%. These extreme values are due to the presence of two highly divergent groups of individuals within the nominal species. Separating the two lineages, divergence within *L. terrestris* s.s is 3.37%, and within *L. herculeus* it is 1.54% (Table 3). Comparing the distribution of the *L. terrestris* intraspecific divergences to what is exhibited among the other species in the genus (Figure 1) we see clearly that the divergence between the two groups found in nominal *L. terrestris* is comparable to the distances among other species of the genus. The mean interspecific divergence between the two *L. terrestris* lineages is 17.5%.

**Table 3:** Kimura 2-parameter mean genetic distances (%) between and within *Lumbricus* spp.

*Lumbricus castaneus*	0.45						
*Lumbricus centralis*	22.99	-					
*Lumbricus festivus*	23.46	18.55	0.36				
*Lumbricus friendi*	22.54	14.80	17.54	-			
*Lumbricus rubellus*	21.04	21.06	21.01	17.72	0.57		
*Lumbricus terrestris*	23.74	18.23	19.82	19.90	18.45	3.37	
*Lumbricus herculeus*	21.62	18.60	17.85	19.43	20.29	17.50	1.54

The principal diagonal has intraspecific distances; all others are interspecific.

**Figure 2:**
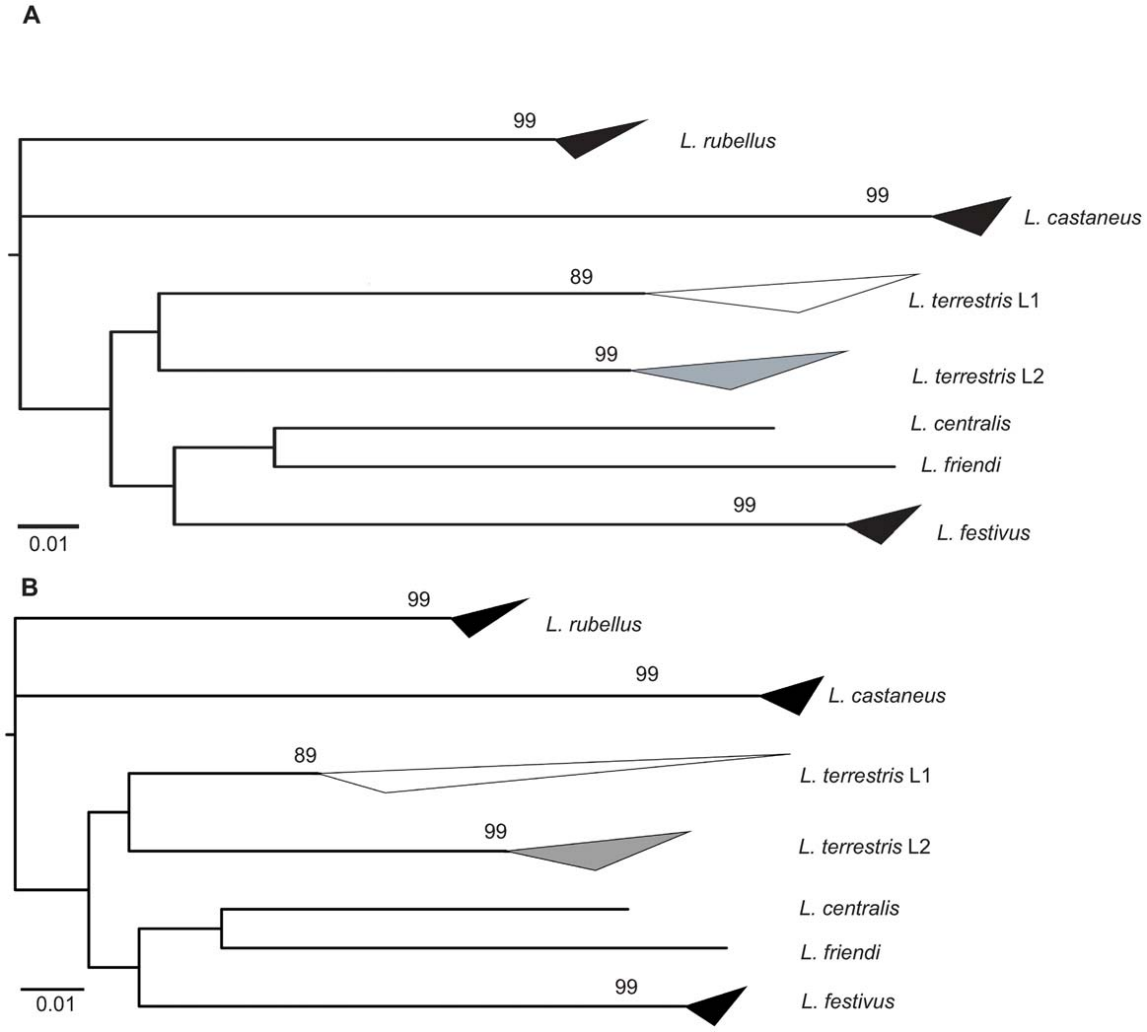
Neighbor joining trees (K2P) for 6 species of the genus *Lumbricus*, based on the COI 5′ ‘barcoding fragment’; bootstrap support values for each cluster shown on its subtending branch. The upper and lower sides of each triangle represent respectively the maximum and minimum genetic distances within a species. A. Without type or museum material of the two *L. terrestris* lineages. B. Reconstruction with type specimens and museum material. Higher genetic variation of *L. terrestris* L1 in B. is due to the short sequence (144bp) of the 1972 museum specimen.

An unrooted Neighbor-Joining (NJ) tree of *Lumbricus* barcode sequences placed all nominal *L. terrestris* in two well-supported and divergent clusters (Figure 2A). All other species represented by more than one individual also fell into well-supported clusters.

In a second step the short barcode sequences from the 1972 museum specimen of *L. terrestris* and from the syntype of *L. herculeus* were introduced in the dataset. Although both the sequences were short, they allowed an accurate assignment of each specimen to one of the *L. terrestris* lineages (Figure 2B). The discriminating power of mini-barcodes is established [13] and here we used these short sequences in favorable conditions as the divergence between the two lineages of *L. terrestris* is very high (17.5%). Thus each of the type-related specimens was successfully assigned to a lineage in the NJ analysis, one to the *L. terrestris* cluster and one to the *L. herculeus* cluster (Figure 2B). From this point forward in the results and discussion, we use the two species names in the restricted sense supported by these data, unless enclosed in quotation marks.

In a separate NJ analysis (tree not shown) the cytochrome oxidase I gene barcode region of the complete “*L. terrestris*” mitochondrial genome sequence [21; GenBank NC001673.1] fell within the *L. terrestris* cluster.

### Morphology

**Figure 3:**
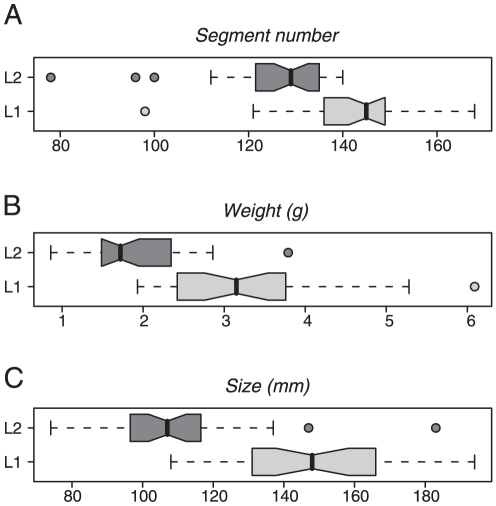
Boxplots of morphological features for *L. terrestris* (L1; N = 30) and *L. herculeus* (L2; N = 36): A. Segment number; B. Body weight; C. Body length. Each boxplot represents: the discarded outliers (external dots), the smallest and largest observations (external bars), the lower and upper quartiles (limits of the box) and the median (within-box black line). The boxplots are notched and indicate that medians differ if the notches do not overlap.

**Figure 4:**
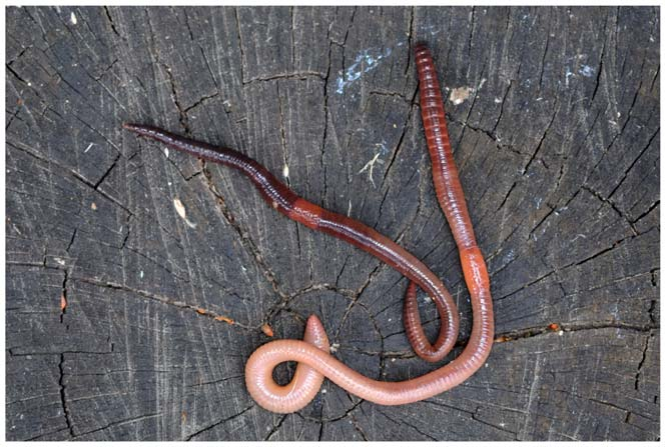
*Lumbricus herculeus* (left) and *Lumbricus terrestris* (right); specimens depicted are respectively smaller and larger than average for their respective species.

Morphological examination of the fresh specimens, each registered in such manner that a COI barcode sequence can be matched to an individual worm, indicates that there are differences in segment number (125 vs 143, *L. herculeus* and *L. terrestris* respectively), body mass (1.7g vs 3.2g), and body length (107mm vs. 148 mm) between the two groups (Figures 3A–C). These differences are not as clear-cut as the genetic differences, there being overlap in the distributions of the three measurements. Put simply, small *L. terrestris* can be smaller than large *L. herculeus*, but they have strongly divergent COI sequences. The two species are illustrated in Figure 4.

The specimen of *L. terrestris* in the vial labeled as neotype (Natural History Museum, London; Register No. 1973.1.1) is shorter by 12 mm and has 6 fewer segments than the neotype described by Sims [8].The other specimens of the same series from Uppsala are all either longer or shorter, or have more or fewer segments than the specimen described in Sims (1973) (E. Sherlock, *in litt.*), so the Sims [8] neotype is presumed lost.

### Geographical distributions

In the Scandinavian material, which covers latitudes from 55 to 68°N, *L. herculeus* was found at seven localities all concentrated in the western part of Sweden's southernmost province, Scania, and at one Danish locality (at Århus in Jutland), the northernmost site being near Båstad, Sweden (56°23′N), while *L. terrestris* was found scattered over Sweden (to Västerbotten in the north, at about 64°N) and also above the Polar Circle on the Norwegian West coast (Nordland, at 68.7°N). All our North American records belonged to *L. terrestris*, while French records were mixed. Two Swedish sites, the Danish site and three French sites had both species, while others had only one.

### Neotypes

We turn now to the means of defining these two very similar species. As we have indicated, size is the only morphological difference, and it is not reliable in the overlapping sections of the size distributions. Above we noted that the neotype of *L. terrestris* collected at Uppsala in 1972 is no longer in the Natural History Museum (London). Neotypes can be replaced “…when no name-bearing type specimen (i.e. holotype, lectotype, syntype or prior neotype) is believed to be extant and an author considers that a name-bearing type is necessary to define the nominal taxon objectively.” [22, Art. 75.1].

If the designation of a new neotype would only replace one morphologically undefinitive specimen with another, then there is little or no justification for the designation. However, we have successfully isolated and sequenced some DNA from a contemporary member of the same population (GenBank HQ024541) as the missing neotype. We also obtained sequence data from specimen CE6377M collected at the same location (GenBank HM388349; BOLD EW-ECO-0533). Then we unequivocally clustered these resulting sequences with those of numerous other individuals which by size are generally identifiable as *L. terrestris*. We also demonstrated a substantial genetic difference between the *L. terrestris* cluster and the related and cryptic congener *L. herculeus*.

Designation of a neotype must demonstrate exceptional need. We believe this to be the case. All of the points raised by Sims [8] regarding stability of nomenclature are still valid today. As Sims [8] indicated in his arguments for the designation of a neotype, *L. terrestris* occupies not only an important historical position in the nomenclature of earthworms as the first earthworm described and type species of *Lumbricus* and the Lumbricidae, it has also been a model organism for research and education in Biology. It is of considerable importance for the stability of nomenclature that it be possible to determine the identity of an earthworm matching the physical characters of *L. terrestris* and *L. herculeus*. Our results indicate that morphological examinations are not sufficient for the identification of these species, but that DNA sequences are. We have established the utility of the barcode fragment of the COI gene [23], [24] for this purpose but do not confine the method to this gene. The proposed neotype now has a COI sequence tag which can unequivocally be used to characterize and recognize the taxon *L. terrestris* in a way hitherto impossible. To date there are no known *Lumbricus* species with a sufficiently similar sequence to cause any confusion in DNA-based identification of species within this genus, let alone in the discrimination of *L. terrestris* and the morphologically virtually identical *L. herculeus*.

To satisfy the provisions of ICZN [22] Art. 75.3.1-7, the qualifying conditions for validly designating a neotype, we offer the following points:

1. Our designation of a neotype is necessary to clarify the taxonomic status of *L. terrestris*, in order that it can be distinguished from *L. herculeus*.
2. The characters differentiating *L. terrestris* from *L. herculeus* are differences in aligned, positionally homologous COI gene DNA sequence bases.
3. The partial COI sequence derived from the neotype (GenBank HM388349; BOLD EW-ECO-0533), and the physical description in Sims [8], which is identical in all but measurements to the designated neotype, and the measurements given here below are sufficient to ensure correct recognition of the specimen designated.
4. The prior neotype is missing from the collection of the Natural History Museum (London) and the staff made a thorough search of the premises, but failed to locate the specimen.
5. Other than the particular body size and segment number measurements, the designated specimen is anatomically consistent with the prior neotype.
6. One of the collectors of the prior neotype (Tryggve Persson) was consulted on the collection event location of 13 October 1972, and the new neotype was taken from the same locality in the Botanical Gardens at Uppsala, Sweden. The location was chosen because that was the location satisfying this condition (proximity to original collection site of Linnaeus) for the prior neotype. We follow this established precedent, which is in any case the valid type locality following the designation of the prior neotype.
7. The new neotype has been deposited in the Swedish Museum of Zoology, with catalogue number given below.

Our choice of neotype specimen is not one of the specimens collected at the same time as the prior neotype, even though we were able to obtain a short (144 bp) sequence of the COI gene from one of three attempted. Therefore regarding “Recommendation 75A. Choice of neotypes.,” [22] we designate a new specimen preserved in a manner that allows extraction of high-quality DNA. Thus the definition and delimitation of the taxon need not be based only on the short “mini-barcode” obtained from the 1972 specimen, because future researchers will be able to use small samples of the newly designated neotype for further genetic data. In short, we maintain that for purposes of molecular definition and delimitation of the taxon, the 1972 material is in poor condition.

The following synonymy is modified from: http://earthworms.elte.hu/Hungary/lumbricus.htm by removal of the references to *L. herculeus*, which was included by Cs. Csuzdi in the synonymy of *L. terrestris*. In all cases where the authors in the following synonymy did not make any distinction between *L. terrestris* and *L. herculeus*, we indicate that the *L. terrestris* referred to may be partly attributable to another species. However we make no claims about the identities of any of the other junior synonyms.

***Lumbricus terrestris*** Linnaeus, 1758

*Lumbricus terrestris*: Linnaeus, 1758 Systema Naturae, 10: 647.

*Lumbricus agricola* Hoffmeister, 1842 Verm. Lumbric., p. 24.

*Lumbricus infelix* Kinberg, 1867 Öfv. Akad. Förh., 23: 98.

*Lumbricus americanus* Perrier, 1872 N. Arch. Mus. Paris, 8: 44.

*Lumbricus terrestris* (part.): Örley 1885 Értek. term. tud. köréből, 15: 30.

*Lumbricus studeri* Ribaucourt, 1896 Rev. suisse Zool., 4: 5.

*Lumbricus terrestris* (part.): Michaelsen 1900 Das Tierreich, 10: 511.

*Lumbricus terrestris* (part.): Szüts 1909 Állattani Közlemények, 8: 142.

*Lumbricus terrestris* (part.): Zicsi 1959 Acta zool. hung., 5: 433.

*Lumbricus terrestris* (part.): Zicsi 1968 Opusc. Zool. Budapest, 8: 130.

*Lumbricus terrestris* (part.): Sims 1973. Bull. Zool. Nom. 30:32.

*Lumbricus terrestris* (part.): Zicsi 1982 Acta zool. hung., 28: 443.

*Lumbricus terrestris* (part.): Easton 1983 Earthworm Ecology, p. 482.

*Lumbricus terrestris* (part.): Zicsi 1991 Opusc. Zool. Budapest, 24: 173.

*Lumbricus terrestris* (part.): Mršić 1991 Acad. Sci. Art. Slov. (Hist. Nat.), 31: 481.

*Lumbricus terrestris terrestris* (part.): Qiu & Bouché 2000 Doc. pedozool. integrol, 4: 192.

*Neotype*. Clitellate specimen. Sweden, Uppland, Uppsala, Uppsala Botanical Garden, walkway adjacent to lawn, 59°51.07′N, 017°37.65′E . 04 June 2009. Collector: Christer Erséus. Swedish Museum of Natural History (Stockholm) catalogue number SMNH Type- 8035.

Other material: 4 clitellates, Sweden, Uppland, Uppsala, Uppsala Botanical Garden,, 13 October 1972. B. Axelsson, U. Lohm, T. Persson collectors; 1 clitellate, France, Parc du Château de Versailles, Ile de France 48° 43′43.52″ N, 2° 06′56.74″ 1 November 2008, M. Hedde collector; 2 clitellates, France, Essone, Ile de France, Parc du Château de Brunoy, 48° 48′41.61″ N, 2° 29′38.25″ 11 November 2008, M. Hedde and T. Decaëns collectors.

Description of neotype and other material: The neotype is in two fragments, the anterior consisting of 59 segments and the posterior of 95 segments, for a total of 154 segments, with total length (strongly contracted) 89 mm. There are herniations on the right side at 15/16 and 28/29, and slight abrasions to the left side of segments 46–52. This damage was present on the specimen at the time of collection from a walkway. The clitellum is at 32–37, the tubercula pubertatis at 33–36, and there are genital markings surrounding enlarged AB genital setae on segments 31–37 and right side of segment 38. The first muscular septum is always 19/20, which is displaced about a half-segment length posteriorly to lie close to septum 20/21.

Examining the 1972 and the French material, the typhlosolar convolutions are very distinctive. From the beginning of the typhlosole in XXII it has lateral flaps oriented vertically. The ventral edges of each flap bifurcate and fuse with the split sections of the flaps anterior and posterior to the flap in question. The fused parts form a short bar extending across the center ventral face of the typhlosole to meet the lateral flaps of the other side, which are also split and fused as just described. The short bars take the appearance of the rungs of a ladder whose lengthwise components are made of the fusion points of the lateral flaps. This pattern originates in segments 23–24 and gradually fades out over two or three segments between 47 and 52, after which the typhlosole has a smooth surface and a circular to oval cross-section. The typhlosole ends abruptly over one or two segments anywhere from 99 to 117, though most commonly in 100–108.

*Remarks*: The neotype and the 1972 specimens agreed in all other particulars with the description in Sims [8], except for the muscularity of septa behind the gizzard. It is possible that Sims [8] mistook the two closely-spaced septa for one and misjudged the count because of the displacement. To be certain, we counted anteriorly from segment 25, where septum 24/25 is in line with its external segment boundary. No morphological differences from the nominal *L. terrestris* as traditionally defined were detected. The sequences given (Genbank HQ024541, HM388349) are the only present means of objective determination of the species, in relation to the slightly smaller *L. herculeus*.

***Lumbricus herculeus*** Savigny, 1826 (synonymy modified from http://earthworms.elte.hu/Hungary/lumbricus.htm)

*Enterion herculeum*: Savigny, 1826 Mem. Acad. Sci. Inst. Fr., 5: 180.

*Lumbricus terrestris* (part.): Örley 1885 Értek. term. tud. köréből, 15: 30.

*Lumbricus terrestris* (part.): Michaelsen 1900 Das Tierreich, 10: 511.

*Lumbricus terrestris* (part.): Szüts 1909 Állattani Közlemények, 8: 142.

*Lumbricus terrestris* (part.): Pop 1943 Ann. Hist.-Nat. Mus. Hung., 36: 19.

*Lumbricus terrestris* (part.): Zicsi 1959 Acta zool. hung., 5: 433.

*Lumbricus terrestris* (part.): Bouché 1972 Inst. Nat. Rech. Agron. p. 352.

*Lumbricus terrestris* (part.): Sims 1973. Bull. Zool. Nom. 30:32.

*Lumbricus terrestris* (part.): Zicsi 1968 Opusc. Zool. Budapest, 8: 130.

*Lumbricus terrestris* (part.): Zicsi 1982 Acta zool. hung., 28: 443.

*Lumbricus terrestris* (part.): Easton 1983 Earthworm Ecology, p. 482.

*Lumbricus terrestris* (part.): Zicsi 1991 Opusc. Zool. Budapest, 24: 173.

*Lumbricus terrestris* (part.): Mršić 1991 Acad. Sci. Art. Slov. (Hist. Nat.), 31: 481.

*Lumbricus terrestris terrestris* (part.): Qiu & Bouché 2000 Doc. pedozool. integrol, 4: 192.

*Lectotype*. Clitellate specimen. In the general area of Paris France. 1821. Collector J.C. Savigny. Paris, Musee Nationale d'histoire Naturelle. Label data: *Enterion herculeum* Savigny, Paris 1821.

Other material: 4 clitellates, France, Parc du Château de Versailles, Ile de France 48° 43′43.52″ N, 2° 06′56.74″ 1 November 2008, M. Hedde collector; 4 clitellates, France, Essone, Ile de France, Parc du Château de Brunoy, 48° 48′41.61″ N, 2° 29′38.25″ 11 November 2008, M. Hedde and T. Decaëns collectors.

Lectotype: Three fragments in one vial, consisting of the first 22 segments, segments 23–50, and segments 51 to 145. There is a partial cut in the 5^th^ segment and a small knotted thread is inserted in the 9^th^ segment, in the manner of those used for tagging larger animal specimens. Total length, 114 mm. Genital markings are present on AB of 9–11, left 26, 31–38, and left 39. The clitellum is saddle-shaped on 32–37, with tubercula pubertatis on 33–36. No pigmentation is visible. No dissection was performed on this delicate specimen. The 480 bp DNA barcode sequence from this specimen is GenBank HQ024540.

The other material examined had no differences from the *L. terrestris* specimens other than measurements (Figs 3A–C., which include additional specimens to those examined in detail). Septal muscularity, and typhlosole morphology and termination were all indistinguishable other than a slight tendency of *L. herculeus* specimens to have fewer atyphlosolate segments than *L. terrestris*. However these numbers overlapped, like the other quantitative measures.

Savigny did not designate any type specimen(s), so here we defined this species in the most simple and direct manner possible at this time. The somewhat softened clitellate specimen was not subjected to further examination, and is now designated as the lectotype of *L. herculeus*. The description of *L. terrestris* by Sims [8] serves as a source of morphological details. Our Figures 3A–C, the observations on the specimens examined for the above descriptions, and the descriptive data of Bouché and Beugnot [25] give the extent of morphological differences between this species and *L. terrestris*.

## Discussion

These results indicate that “*L. terrestris*” as traditionally identified is composed of two species that have not been discriminated in the literature. Morphological examination can only make reliable distinctions between average or larger *L. terrestris* and average or smaller *L. herculeus*. Bouché and Beugnot [25] reached the same conclusion regarding what they considered as two sympatric populations of “*L. herculeus*” with nomenclature following Bouché's 1970 advocacy of that name over “*L. terrestris.*” Our collection includes 18 individuals from the Parc du Chateau de Brunoy location sampled by Bouché and Beugnot (1972), of which 16 fall in *L. herculeus* and the other 2 in *L. terrestris*. The segment number and size variations Bouché and Beugnot [25] reported are the same as we observed between *L. herculeus* and *L. terrestris*. The two species are best distinguished by molecular data, which will work on all sizes and life stages of the individuals, from egg capsules to adults. The sequences from 1972 and 2009 specimens topotypic to the missing Sims [8] *L. terrestris* neotype were in the *L. terrestris* cluster.

In a similar situation, that of earthworms questionably separable by size, body coloration, and some genital papillae, Chang et al. [26] found that molecular data strongly supported separation of two species from nominal *Amynthas wulinensis* Tsai, Shen and Tsai, 2001. In the *A. wulinensis* case, the size, color, and papillae characters giving the initial indications of lineage diversity are traditionally not considered reliable in Asian earthworm taxonomy. *Eisenia fetida* (Savigny, 1826) and *E. andrei* Bouché, 1972 are only sometimes separable by color, but are two genetically distinct and isolated species [27].

Savigny [9] did not attribute an author to *Enterion herculeum* and did not indicate that it was a new species, which was not required in his time. Nor did he expressly indicate that *Enterion herculeum* is a replacement name for some other nominal species group [25, Art. 72.7]. Therefore the two names are not objective synonyms, and *L. herculeus* is a junior subjective synonym of *L. terrestris*. In any case the brief description by Savigny is a valid indication [25, Art. 12.2.] of the identity of the worm and intent of the author. The effect of our work is to restore a junior subjective synonym (*L. herculeus*) to species status. In consideration of the molecular data and nomenclatural procedure, we remove *L. herculeus* from the synonymy of *L. terrestris*, and thereby restrict *L. terrestris* to the cluster whose members are larger.

Apparently, Savigny was describing a new species, named *Enterion herculeum*, among 21 other names in his document. Had his specimens been *L. terrestris* s.s., the name *herculeus* would definitely pass into synonymy. By happenstance, he collected and applied a name to what is now defined as a separate species. Where Linnaeus worked in Sweden he saw earthworms on the surface at night [1: p. 648: “*ascendit noctu*”]. Sweden has both species, with *L. herculeus* only found in Scania so far, but regardless of which species Linnaeus saw, *L. terrestris* was defined by the Sims [8] neotype and is now defined by its replacement. On the other side of the Atlantic, the brisk trade in fish bait and classroom specimens is so far known to consist only of *L. terrestris*. North American investigators may rely on the lack of records of *L. herculeus*, but do so at their peril. We would not be surprised to find *L. herculeus* in North America or other continents where “*L. terrestris*” is known to occur.

This revision introduces doubt about the true identity of the species involved in any publication on “*L. terrestris*”, even if vouchers were deposited. Larger *L. terrestris* can be fairly certainly identified, as can smaller *L. herculeus*. Otherwise, old vouchers fixed in un-buffered formaldehyde solutions may not yield usable DNA, and therefore may not be identifiable. However, the worm used in Boore and Brown [21] for a complete mitochondrial genome sequence (GenBank NC001673.1) was apparently *L. terrestris*.

At this point it is an open question whether or not research on these two highly similar species has been tainted by the taxonomic confusion of the last 200 years. Are there conflicting results from similar studies, which could be resolved by establishing the true identities of the earthworms involved? Here we do not speak of the instances where careless study of “the earthworm, *Lumbricus terrestris*” actually referred to some other species, but to those in which a perfectly honest error was made, because taxonomists had no access to the types of data necessary to make the distinctions we are making here, and are now easily obtained. The two species seldom co-occur in northern France, which could be due to competitive exclusion in various habitats that favor one or the other species. Alternatively they could be different enough to have distinct habitat preferences, and seldom come into competition. All northern European populations have been established by human-aided and natural dispersal since the retreat of the last European ice sheet. Thus we are not speaking of natural allopatric distributions but of a combination of accidents of arrival and competitive exclusion [28].

The obvious consequence of the revision is that any future identification of *Lumbricus* species closely resembling *L. terrestris* and *L. herculeus* should be accomplished in part by comparing DNA sequences including the COI barcode region. The genetic “gap” between the two is large and there are no known intermediate populations, so the results should be very clear.

## Supporting Information

Table S1 (DOC)
