# Supplementary material for: DNA Barcoding Reveals Cryptic Diversity in Lumbricus terrestris L., 1758 (Clitellata): Resurrection of L. herculeus (Savigny, 1826)
Source: PLoS One. 2010 Dec 29;5(12):e15629. doi: 10.1371/journal.pone.0015629 (PMC3012069; doi:10.1371/journal.pone.0015629)
Supplement: Table S1 — (DOC) [file pone.0015629.s001.doc]

| **Genbank Number** | **Identification** | **Genbank Number** | **Identification** | **Genbank Number** | **Identification** |
| --- | --- | --- | --- | --- | --- |
| GU206160 | Lumbricus castaneus | HQ024636 | Lumbricus terrestris | HM388349 | Lumbricus terrestris |
| GU206156 | Lumbricus castaneus | HQ024581 | Lumbricus terrestris | HQ024550 | Lumbricus terrestris |
| GU206157 | Lumbricus castaneus | HQ024627 | Lumbricus terrestris | HQ024549 | Lumbricus terrestris |
| FJ937284 | Lumbricus castaneus | HQ024580 | Lumbricus terrestris | HQ024548 | Lumbricus terrestris |
| GU206152 | Lumbricus castaneus | HQ024626 | Lumbricus terrestris | HQ024547 | Lumbricus terrestris |
| GU206153 | Lumbricus castaneus | HQ024625 | Lumbricus terrestris | HQ024546 | Lumbricus terrestris |
| GU206154 | Lumbricus castaneus | HQ024579 | Lumbricus terrestris | HM388350 | Lumbricus terrestris |
| GU013842 | Lumbricus castaneus | HQ024624 | Lumbricus terrestris | HQ024545 | Lumbricus terrestris |
| GU206155 | Lumbricus castaneus | HQ024623 | Lumbricus terrestris | HQ024544 | Lumbricus terrestris |
| GU206159 | Lumbricus castaneus | GU014230 | Lumbricus terrestris | HQ024543 | Lumbricus terrestris |
| GU013982 | Lumbricus centralis | GU206223 | Lumbricus terrestris | HM388351 | Lumbricus terrestris |
| FJ937288 | Lumbricus festivus | GU014224 | Lumbricus terrestris | HM388352 | Lumbricus terrestris |
| FJ937294 | Lumbricus festivus | FJ937323 | Lumbricus terrestris | HM388353 | Lumbricus terrestris |
| FJ937293 | Lumbricus festivus | HQ024622 | Lumbricus terrestris | HQ024542 | Lumbricus terrestris |
| FJ937302 | Lumbricus festivus | FJ937322 | Lumbricus terrestris | HQ024650 | Lumbricus terrestris |
| HQ024537 | Lumbricus festivus | HQ024621 | Lumbricus terrestris | HQ024649 | Lumbricus terrestris |
| FJ937289 | Lumbricus festivus | FJ937308 | Lumbricus terrestris | HQ024648 | Lumbricus terrestris |
| FJ937286 | Lumbricus festivus | FJ937321 | Lumbricus terrestris | HQ024647 | Lumbricus terrestris |
| FJ937291 | Lumbricus festivus | HQ024578 | Lumbricus terrestris | HQ024646 | Lumbricus terrestris |
| FJ937303 | Lumbricus festivus | FJ937311 | Lumbricus terrestris | HQ024645 | Lumbricus terrestris |
| GU014034 | Lumbricus friendi | GU206238 | Lumbricus terrestris | HQ024644 | Lumbricus terrestris |
| GU206175 | Lumbricus rubellus | HQ024577 | Lumbricus terrestris | HQ024643 | Lumbricus terrestris |
| HQ024539 | Lumbricus rubellus | FJ937324 | Lumbricus terrestris | HQ024642 | Lumbricus terrestris |
| GU206172 | Lumbricus rubellus | HQ024620 | Lumbricus terrestris | HQ024641 | Lumbricus terrestris |
| GU206190 | Lumbricus rubellus | HQ024576 | Lumbricus terrestris | HQ024671 | Lumbricus herculeus |
| GU206178 | Lumbricus rubellus | HQ024607 | Lumbricus terrestris | HQ024670 | Lumbricus herculeus |
| GU206180 | Lumbricus rubellus | HQ024619 | Lumbricus terrestris | HQ024669 | Lumbricus herculeus |
| GU206173 | Lumbricus rubellus | FJ937310 | Lumbricus terrestris | HQ024668 | Lumbricus herculeus |
| GU206190 | Lumbricus rubellus | GU206224 | Lumbricus terrestris | FJ937318 | Lumbricus herculeus |
| HQ024538 | Lumbricus rubellus | GU206217 | Lumbricus terrestris | HQ024667 | Lumbricus herculeus |
| HQ024606 | Lumbricus terrestris | HQ024575 | Lumbricus terrestris | FJ937317 | Lumbricus herculeus |
| HQ024634 | Lumbricus terrestris | FJ937307 | Lumbricus terrestris | GU206237 | Lumbricus herculeus |
| HQ024605 | Lumbricus terrestris | HQ024574 | Lumbricus terrestris | FJ937316 | Lumbricus herculeus |
| GU206215 | Lumbricus terrestris | HQ024573 | Lumbricus terrestris | HQ024666 | Lumbricus herculeus |
| HQ024604 | Lumbricus terrestris | FJ937306 | Lumbricus terrestris | FJ937315 | Lumbricus herculeus |
| GU206216 | Lumbricus terrestris | HQ024572 | Lumbricus terrestris | HQ024665 | Lumbricus herculeus |
| HQ024633 | Lumbricus terrestris | FJ937305 | Lumbricus terrestris | HQ024664 | Lumbricus herculeus |
| GU014223 | Lumbricus terrestris | HQ024571 | Lumbricus terrestris | HQ024663 | Lumbricus herculeus |
| HQ024603 | Lumbricus terrestris | HQ024570 | Lumbricus terrestris | FJ937314 | Lumbricus herculeus |
| HQ024632 | Lumbricus terrestris | HQ024618 | Lumbricus terrestris | FJ937313 | Lumbricus herculeus |
| HQ024602 | Lumbricus terrestris | HQ024569 | Lumbricus terrestris | HQ024662 | Lumbricus herculeus |
| HQ024601 | Lumbricus terrestris | HQ024617 | Lumbricus terrestris | FJ937312 | Lumbricus herculeus |
| HQ024600 | Lumbricus terrestris | GU206225 | Lumbricus terrestris | HQ024661 | Lumbricus herculeus |
| HQ024599 | Lumbricus terrestris | HQ024568 | Lumbricus terrestris | HQ024660 | Lumbricus herculeus |
| FJ937309 | Lumbricus terrestris | HQ024616 | Lumbricus terrestris | FJ937304 | Lumbricus herculeus |
| HQ024598 | Lumbricus terrestris | HQ024615 | Lumbricus terrestris | HQ024659 | Lumbricus herculeus |
| HQ024597 | Lumbricus terrestris | HQ024614 | Lumbricus terrestris | HQ024658 | Lumbricus herculeus |
| HQ024596 | Lumbricus terrestris | HQ024567 | Lumbricus terrestris | GU206234 | Lumbricus herculeus |
| HQ024595 | Lumbricus terrestris | HQ024566 | Lumbricus terrestris | GU206239 | Lumbricus herculeus |
| HQ024594 | Lumbricus terrestris | HQ024565 | Lumbricus terrestris | HQ024657 | Lumbricus herculeus |
| HQ024593 | Lumbricus terrestris | HQ024564 | Lumbricus terrestris | GU206233 | Lumbricus herculeus |
| HQ024592 | Lumbricus terrestris | HQ024563 | Lumbricus terrestris | GU206235 | Lumbricus herculeus |
| HQ024591 | Lumbricus terrestris | HQ024562 | Lumbricus terrestris | GU206236 | Lumbricus herculeus |
| HQ024590 | Lumbricus terrestris | HQ024561 | Lumbricus terrestris | GU206227 | Lumbricus herculeus |
| FJ937295 | Lumbricus terrestris | HQ024560 | Lumbricus terrestris | GU206228 | Lumbricus herculeus |
| HQ024589 | Lumbricus terrestris | HQ024635 | Lumbricus terrestris | GU206229 | Lumbricus herculeus |
| GU206218 | Lumbricus terrestris | HQ024613 | Lumbricus terrestris | GU206230 | Lumbricus herculeus |
| HQ024588 | Lumbricus terrestris | HQ024559 | Lumbricus terrestris | GU206231 | Lumbricus herculeus |
| HQ024587 | Lumbricus terrestris | HQ024612 | Lumbricus terrestris | GU206232 | Lumbricus herculeus |
| GU206219 | Lumbricus terrestris | HQ024558 | Lumbricus terrestris | FJ937300 | Lumbricus herculeus |
| HQ024586 | Lumbricus terrestris | HQ024611 | Lumbricus terrestris | HQ024656 | Lumbricus herculeus |
| GU206222 | Lumbricus terrestris | HQ024557 | Lumbricus terrestris | FJ937299 | Lumbricus herculeus |
| HQ024640 | Lumbricus terrestris | HQ024610 | Lumbricus terrestris | HQ024655 | Lumbricus herculeus |
| FJ937325 | Lumbricus terrestris | HQ024556 | Lumbricus terrestris | HQ024654 | Lumbricus herculeus |
| HQ024585 | Lumbricus terrestris | HQ024555 | Lumbricus terrestris | FJ937297 | Lumbricus herculeus |
| GU206220 | Lumbricus terrestris | GU206226 | Lumbricus terrestris | HQ024653 | Lumbricus herculeus |
| HQ024631 | Lumbricus terrestris | HQ024609 | Lumbricus terrestris | FJ937296 | Lumbricus herculeus |
| GU206221 | Lumbricus terrestris | GU014229 | Lumbricus terrestris | HQ024652 | Lumbricus herculeus |
| HQ024630 | Lumbricus terrestris | HQ024554 | Lumbricus terrestris | HQ024651 | Lumbricus herculeus |
| FJ937320 | Lumbricus terrestris | GU014228 | Lumbricus terrestris | HQ024540 | Lumbricus herculeus |
| HQ024584 | Lumbricus terrestris | HQ024608 | Lumbricus terrestris |  | |
| HQ024583 | Lumbricus terrestris | GU206213 | Lumbricus terrestris |  | |
| HQ024638 | Lumbricus terrestris | GU014227 | Lumbricus terrestris |  | |
| HQ024639 | Lumbricus terrestris | GU014226 | Lumbricus terrestris |  | |
| HQ024637 | Lumbricus terrestris | HQ024553 | Lumbricus terrestris |  | |
| GU014231 | Lumbricus terrestris | HQ024552 | Lumbricus terrestris |  | |
| HQ024629 | Lumbricus terrestris | GU206214 | Lumbricus terrestris |  | |
| FJ937319 | Lumbricus terrestris | GU014225 | Lumbricus terrestris |  | |
| HQ024628 | Lumbricus terrestris | HQ024551 | Lumbricus terrestris |  | |
| HQ024582 | Lumbricus terrestris | HQ024541 | Lumbricus terrestris |  | |
